# Virulence factors, prevalence and potential transmission of extraintestinal pathogenic *Escherichia coli* isolated from different sources: recent reports

**DOI:** 10.1186/s13099-019-0290-0

**Published:** 2019-02-21

**Authors:** Jolanta Sarowska, Bozena Futoma-Koloch, Agnieszka Jama-Kmiecik, Magdalena Frej-Madrzak, Marta Ksiazczyk, Gabriela Bugla-Ploskonska, Irena Choroszy-Krol

**Affiliations:** 1 Department of Basic Sciences, Faculty of Health Sciences, Wroclaw Medical University, Chalubinskiego 4, 50-368 Wroclaw, Poland; 2 Department of Microbiology, Institute of Genetics and Microbiology, University of Wroclaw, Przybyszewskiego 63/77, 51-148 Wroclaw, Poland

**Keywords:** *Eschericha coli*, Virulence, ExPEC, Adhesin, Toxin, Siderophore

## Abstract

Extraintestinal pathogenic *E*. *coli* (ExPEC) are facultative pathogens that are part of the normal human intestinal flora. The ExPEC group includes uropathogenic *E. coli* (UPEC), neonatal meningitis *E. coli* (NMEC), sepsis-associated *E. coli* (SEPEC), and avian pathogenic *E. coli* (APEC). Virulence factors (VF) related to the pathogenicity of ExPEC are numerous and have a wide range of activities, from those related to bacteria colonization to those related to virulence, including adhesins, toxins, iron acquisition factors, lipopolysaccharides, polysaccharide capsules, and invasins, which are usually encoded on pathogenicity islands (PAIs), plasmids and other mobile genetic elements. Mechanisms underlying the dynamics of ExPEC transmission and the selection of virulent clones are still poorly understood and require further research. The time shift between colonization of ExPEC and the development of infection remains problematic in the context of establishing the relation between consumption of contaminated food and the appearance of first disease symptoms. What appears to be most difficult is to prove that ExPEC strains cause disease symptoms and to examine the mechanism of transition from the asymptomatic colonization of the intestines to the spreading of the bacteria outside the digestive system. A significant problem for researchers who are trying to ascribe ExPEC transmission to food, people or the environment is to draw the distinction between colonization of ExPEC and infection. Food safety is an important challenge for public health both at the production stage and in the course of its processing and distribution. Examination of the genetic similarity of ExPEC strains will allow to determine their origin from different sources. Many levels of genotyping have been proposed in which the typing of strains, plasmids and genes is compared in order to obtain a more complete picture of this complex problem. The aim of our study was to characterize *E. coli* strains isolated from humans, animals and food for the presence of bacterial genes encoding virulence factors such as toxins, and iron acquisition systems (siderophores) in the context of an increasing spread of ExPEC infections.

## Introduction

*Escherichia coli* are a group of bacteria normally found in the flora of human and animal digestive tracts and symbionts participating in digestion and synthesis of certain vitamins. Currently, 171 somatic (O), 55 flagellar (H) and 80 capsular (K) antigens have been identified, and there are over 160 serological types of *E. coli*. *E. coli* are involved in the urinary tract infection (UTI), hospital-acquired pneumonia (HAP), sepsis, surgical site infection (SSI), gastrointestinal tract infections, hemolytic-uremic syndrome (HUS), meningitis and inflammation of the meninges [1].

Mobile genetic elements of *E. coli* can be horizontally exchanged in related bacteria or in bacteria from different families, which allows their settlement in different environments. *E. coli* strains can be classified into the following phylogenetic groups: A, B1, B2, C, D, E, F, and clade I [2, 3]. Commensal *E. coli*, with no pathogenic features, that occur, among others, on the gastrointestinal tract mucosa, most often represent group A or B1. Pathogenic *E. coli* responsible for intestinal infections represent phylogenetic groups A, B1 or D. *E. coli* responsible for extraintestinal infections belong to groups B2 and D. Group E is related to group D (including O157: H7), while group F is related to the main group B2. Clones of *E. coli* strains, which are genetically diverse but phenotypically indistinguishable, have been assigned to cryptic clade I [4, 5].

There are some studies that revealed that the avian pathogenic *E. coli* (APEC) and extraintestinal pathogenic *E. coli* (ExPEC) strains, that cause infections in humans, are quite closely phylogenetically related and share some of the same virulence genes [6, 7]. It is possible that APEC strains could hypothetically pose a health risk to humans [8]. A genetic analysis carried out by Rodriguez-Siek and coworkers showed that APEC strains could be a reservoir of virulence genes of ExPEC, pathogenic to humans. This could be the reason for the genetic diversity and genes exchange among pathogenic *E. coli* strains. Some of human extraintestinal pathogenic *E. coli* strains have the *iss* gene in their genomes, which is responsible for increased survival of bacteria in the serum. The *iss* gene is located on plasmid ColV, a huge virulence plasmid typical of avian pathogenic *E. coli* strains, which indicates that an exchange of plasmids and, consequently, exchange of those virulence genes is possible between human and avian pathogenic *E. coli* strains [6].

The development of techniques of molecular biology enables for sequencing whole genomes of reference *E. coli* strains for example: commensal *E. coli* K-12, pathogenic strain O157:H7 that causes intestinal infections and uropathogenic *E. coli* J96. There are also known whole genetic sequences of at least 20 *E. coli* strains [2, 9]. Analysis of sequences of house-keeping genes that is MutliLocus Sequence Typing, MLST made possible to more accurate exploration the phylogenetic structure of *E. coli* species [10, 11]. This method based on determining the allel’s types of selected house-keeping genes and strains are assigned to Sequence Types, (STs) [2]. Analysis with MLST application showed faults and blanks in the past division of *E. coli* into four main phylogenetic groups. Analysis with sequencing revealed that among *E. coli* species there are hybrid groups and about 80–85% of *E. coli* strains were incorrectly assigned to phylogenetic groups [11–13]. With application of MLST revealed that among multidrug resistant extraintestinal pathogenic *E. coli* strains the most frequent is Sequence Type 131 (ST131) [14]. ST131 is a clone of *E. coli* disseminated worldwide and resistant to many antibiotics [15].

### ExPEC—the spectrum of diseases

**Fig. 1:**
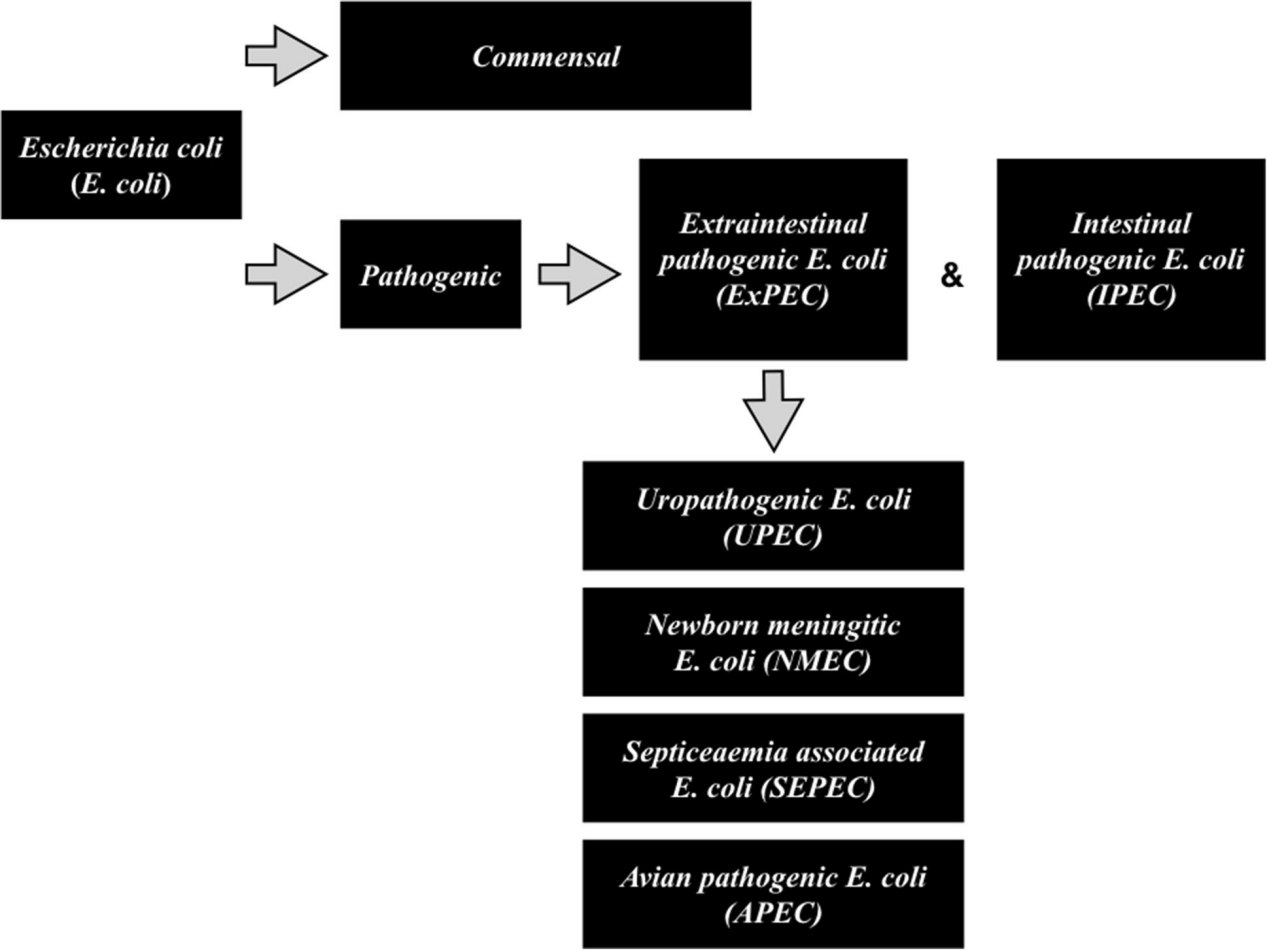
Pathogenic diversity of *Escherichia coli* strains

Extraintestinal pathogenic *E. coli* (ExPEC) (Dale i Woodford) have a complex phylogenetic structure, wide range of virulence factors (VF), and considerable plasticity of the genome. These strains not only cause uncomplicated UTIs, but also bacteremia or sepsis. Mechanisms underlying the dynamics of ExPEC transmission and the selection of resistant clones are still poorly understood and require further research [16]. The ExPEC group includes uropathogenic *E. coli* (UPEC), neonatal meningitis *E. coli* (NMEC), sepsis-associated *E. coli* (SEPEC), and avian pathogenic *E. coli* (APEC) (Table 1, Fig. 1). ExPEC *E. coli* have many virulence-associated factors, including adhesins, toxins, iron acquisition factors, lipopolysaccharides, polysaccharide capsules, and invasins, which are usually encoded on pathogenicity islands (PAIs), plasmids, and other mobile genetic elements [4, 5]. Urinary tract infection is one of the most common infectious diseases. Urinary tract represents a sterile space with the exception of the distal urethra. Urinary tract infections account for approximately 40% of all nosocomial infections and for 10–20% hospital-acquired infections, of which 81% occur in women aged 16–35 years. In men above 60 years of age, the incidence of UTI increases, which is associated with impaired urinary outflow due to enlarged prostate gland [17]. Urinary tract infections affect about 10% of the pediatric population and are diagnosed in about 1% of boys and about 3–8% of girls. In the neonatal period, the infection is more common in boys (approximately 60%), whereas from the age of 2–3 months this tendency is reversed. As there are no characteristic clinical symptoms in this period of life, sometimes the first observed symptoms of UTI are psychosomatic disorders or hypertension [18, 19]. Clinical symptoms associated with UTI may have a different clinical picture, ranging from asymptomatic bacteriuria, various ascending infections (e.g. acute pyelonephritis), up to severe urosepsis [18]. The most common etiologic pathogens associated with UTI include Gram-negative *Enterobacteriaceae*, with predominating strains of uropathogenic *E. coli* [19]. *E. coli* strains are responsible for 75–95% of uncomplicated UTIs and for 40–50% of complicated UTIs [17].

**Table 1:** Virulence genes of ExPEC encoding adhesins, toxins and siderophores

Description	Virulence genes	Function	ExPEC pathotype
*Adhesins*			
Type 1 fimbriae	*fim*	Factor of colonization in extraintestinal infections, biofilm formation	UPEC, NMEC, SEPEC, APEC
Afimbrial adhesin	*afa*	The non-fibrous adhesin binds to the DAF receptor on the cell surface epithelium, hemagglutination capacity	UPEC
Dr fimbriae	*dra*	Binding to the DAF receptor on the surface epithelial cells and mediation of internalization bacteria to the host cells	UPEC
P fimbriae	*pap*	Stimulate the production of cytokines by T lymphocytes, colonization factor in extraintestinal infections	UPEC, SEPEC, APEC
S fimbriae	*sfa*	Adhesion to intestinal epithelial cells, kidney, lower urinary tract cells; facilitate the penetration of bacteria into the tissues	UPEC, NMEC
F1C fimbriae	*foc*	Adhesion to renal epithelial cells and endothelial cells of the bladder and kidneys	UPEC
Iha	*iha*	Iron‐regulated‐gene‐homologue adhesion	UPEC
Mat	*mat*	Meningitis associated and temperature regulated fimbriae	NMEC
Curli fiber gene	*crl, csg*	Enable biofilm formation and promote pathogenicity	UPEC, SEPEC, APEC
Antigen43	*agn43(flu)*	Protein of autotransporter family, adhesion and biofilm development	UPEC
*Invasine*			
Ibe ABC	*ibeA,B,C*	Cell invasion into the host tissues	NMEC, SEPEC, APEC
*Iron uptake*			
Aerobactin	*iuc,aer*	Siderophore, acquisition of Fe2 + / 3 + in the host system	UPEC, APEC
Iron repressible protein	*irp*	Yersiniabactin synthesis	NMEC
Salmochelin	*iroN*	Siderophore receptor, use of Fe ions obtained from the body host	UPEC, NMEC, SEPEC APEC
ChuA, Hma	*chu, hma*	Enable using of Fe from hemoglobin in the host system	UPEC, SEPEC
SitABC	*sitA,B,C*	Transportation of Fe, Mn	UPEC, APEC
*Protectins/serum resistance*			
Transfer protein	*traT*	Inhibition of the classical pathway of complement activity	NMEC, SEPEC APEC
Capsula antigens	*KpsMI-neuA, KpsMII*	The protection factor against phagocytosis and the spreading factor	NMEC, SEPEC
Outer membrane protein	*omp*	Enable intracellular survival, evasion from the body’s defense.	UPEC, NMEC
Increased serum survival	*iss*	The protection factor against phagocytosis	NMEC, SEPEC, APEC
ColV, CvaC	*colV, cvaC*	Factor facilitating colonization	NMEC, SEPEC, APEC
*Toxins*			
Serin protease autotransporter	*pic*	Degrades mucins, facilitates colonization epithelium, damages of the cell membrane	UPEC
Secreted autotransporter toxin	*sat*	Proteolytic toxin, effect cytotoxic—influences on cell vacuolization	UPEC
Vacuolating autotransporter toxin	*vat*	Proteolytic toxin, induces host cell vacuolization	UPEC, APEC
Hemolysin A	*hlyA*	Creating of pores in membranes of host cells (cell lysis)	UPEC
Cytotoxic necrotizing factor	*cnf*	Engaging in cell necrosis	UPEC, SEPEC
Cytolethal distending toxin	*cdt*	Cytolethal distending factor	SEPEC

ExPEC are facultative pathogens that are part of the normal human intestinal flora, but their presence may be associated with some infectious diseases such as UTIs, neonatal meningitis (NMEC *E. coli*) and sepsis (SEPEC *E. coli*), with UTI being the most prevalent. Most UTIs are caused by a highly heterogeneous group of ExPEC, termed uropathogenic *E. coli* (UPEC). Urinary tract infection is a social problem that affects people all over the world; in the United States, for example, UTIs account for 4–5 billion dollars in health care costs a year [20].

APEC causing colibacillosis in poultry is considered to be a pathotype of ExPEC. Colibacillosis seems to be the major infectious disease in birds, responsible for significant economic losses of poultry farms worldwide, leading to high mortality and a decrease in poultry production [21]. By its association with various disease conditions, APEC can cause infections of extraintestinal organs or sepsis and also secondary infections in poultry and in wildfowl. The diseases that may be caused by APEC in fowl include respiratory tract infections, septicemia, polyserositis, coligranuloma, cellulitis, yolk sac infection, omphalitis, and swollen head syndrome [22].

### ExPEC—the specific virulence factors

Uropathogenic *E. coli* have many virulence factors, i.e. adhesins, toxins (e.g. alpha-hemolysin, cytotoxic necrotizing factor 1, autotransporter toxins), iron/heme-acquisition systems, and iron ion transport. P, S and type 1 fimbriae are responsible, among others, for adhesion to epithelial cells of intestines, kidneys, or lower urinary tract, and for stimulating cytokine production by T cells. Moreover, they are an important colonization factor in extraintestinal infections. A characteristic feature of UPEC is the ability to multiply intracellularly [2, 23]. The loss of a portion of the genome involved in the production of type 1 fimbriae and inactivation of genes encoding P fimbriae have led to the formation of strains responsible for asymptomatic bacteriuria (ABU). These strains can colonize the urinary tract without inducing inflammation [24]. A potential source of UPEC is host own intestinal flora, but the infection can also be transmitted through the fecal–oral route or through sexual contact [24]. *E. coli* K1 strains are the etiological factor of the majority of neonatal invasive infections such as meningitis, bacteremia/sepsis, and the severity of such infections is directly related to the presence and amount of capsular antigen. The potential source of infection with *E. coli* K1 strains may be hospital staff [1]. Early *E. coli* infection may have its source in the urogenital or digestive tract, particularly in the case of UTI in the perinatal period. In 2010, the National Reference Centre for Infections of Central Nervous System (KOROUN) registered 68 cases of invasive *E. coli* infections, including 19 in newborns [1, 25].

The NMEC strains have the ability to survive in the bloodstream and invade meninges of infants. Neonatal meningitis caused by *E. coli* strains is one of the most common infections contributing to high neonatal mortality (10%) and morbidity (30%) rate. A considerable genotypic and phenotypic heterogeneity among NMEC strains poses many difficulties in determining characteristics that can be used to distinguish them from commensal *E. coli* [26]. Important VFs of NMEC include K1 capsular antigen, protecting against phagocytosis and responsible for bacteria spreading, *ibeA*, *B*, *C*, promoting invasion into cells and further into the tissues, Iss protein protecting against phagocytosis—bactericidal action of serum, and the colony stimulating factor V, which is not present in the UPEC strains. The NMEC and SEPEC isolates include *ibeA, B, C, traT, iss, colV, cvaC, gimB*, *sfa/foc* genes encoded on plasmids, which are not found in the studied UPEC isolates [27].

APEC strains are characterized by a specific set of virulence genes which enable the bacteria to cause extraintestinal infections. The virulence factors typical of APEC include haemolysins (HlyE), colicins (CvaC), increased serum survival protein (iss), fimbriae type I (FimC), temperature-sensitive hemagglutinin (Tsh), and siderophores (IucC, SitA) [28, 29].

ExPEC is a major cause of infections, particularly UTIs and septicemia, in both humans and domestic animals. Resistance to oxyimino-cephalosporins (e.g. cefotaxime, ceftazidime, cefovecin and ceftiofur) is often due to the production of hydrolyzing enzymes known as extended-spectrum β-lactamases (ESBLs). Antibiotic resistance in *E. coli* is a rapidly expanding problem due to the organism’s ability to mutate, acquire and transmit plasmids and other mobile genetic elements encoding resistance genes [30].

### ExPEC pathogenicity-associated islands and ExPEC virulence associated plasmids

PAIs are specific regions on the bacterial chromosome where virulence genes accumulate. PAIs and their associated virulence genes spread among bacterial populations by horizontal transfer [31]. Several PAIs have previously been identified in uropathogenic *E. coli* strains such as *E. coli* 536, *E. coli* J96, *E. coli* CFT073. PAIs I to IV from strain 536 encode a range of virulence factors, including P fimbriae, P-related fimbriae, α-hemolysin, S-fimbriae and yersiniabactin siderophore system. PAIs IJ96 and IIJ96 encode P fimbriae, P-related fimbriae and α-hemolysin. PAIs ICFT073 and IICFT073 encode P fimbriae, α-hemolysin and aerobactin [27]. Detailed characteristics of the pathogenicity islands and the function encoded are presented in Table 2. PAIs are frequent among *E. coli* strains causing extraintestinal infections, and ExPEC strains mainly belong to phylogenetic groups B2 and D [32].

**Table 2:** The pathogenicity islands and the functions encoded

Pathogenicity islands or gene	Products
*Pathogenicity islands*	
II_CFT073_	P fimbriae, iron-regulated proteins
I_536_	α-Hemolysin, F-17-like fimbriae, CS12-like fimbriae
II_536_	Hek adhesin, P- related fimbriae, α-Hemolysin, hemagglutinin-like adhesion
III_536_	S fimbriae, salmochelin, HmuR-like heme receptor, Sat toxin, Tsh-like hemoglobin protease, antigen 43
IV_536_	Yersiniabactin siderophore system
II_J96_	α-Hemolysin, Prs fimbriae, cytotoxic, necrotizing factor
*Virulence associated genes*	
*malX*	Maltose- and glucose-specific component IIa of a phosphoenolpuryvate-dependent phosphotransferase system
*usp*	Putative bacteriocin

Naturally occurring plasmids are able to promote the dissemination of a variety of traits including drug resistance, virulence and the metabolism of rare substances. *E. coli* have been found to possess a variety of plasmid types including those associated with virulence. Despite the large number of plasmid types known to occur among *E. coli* strains, plasmids encoding virulence-associated traits fall almost exclusively within a single incompatibility family known as IncF. Like the intestinal pathotypes, many EXPEC strains also contain virulence plasmids [5]. These plasmids are listed and described in Table 3.

**Table 3:** Sequenced ExPEC virulence-associated plasmids

Plasmid	Size (bp)	Replicon (s)	Key component (s)	GenBank accession no. or source
pAPEC-1	103,275	FIB, FIIA	Aerobactin, *sit, hlyF, ompT, ets, iss,* salmochelin, CoIV, *tsh*	CP000836
pAPEC-O103-CoIBM	124,705	FIB, FIIA	Aerobactin, *sit, hlyF, ompT, iss,* salmochelin, CoIBM	CP001232
pECOS88	133,853	FIB, FIIA	Aerobactin, *sit, hlyF, ompT,ets, iss,* salmochelin, CoIV	CU928146
pVM01	151,002	FIB, FIIA	Aerobactin, *sit, hlyF, ompT, iss, ets*, salmochelin, CoIBM, *tsh*	EU330199
pAPEC-O1-CoIBM	174,241	FIB, FIIA	Aerobactin, *sit, hlyF, ompT, iss, ets*, salmochelin, CoIBM, *tsh*	DQ381420
pAPEC-O2-CoIV	184,501	FIB, FIIA	Aerobactin, *sit, hlyF, ompT, ets, iss,* salmochelin, CoIV, *tsh*	AY545598

ExPEC include NMEC, one of the predominant agents of neonatal bacterial meningitis [33]. The virulence-associated genes of the NMEC plasmid core includes all genes of the aerobactin (*iutA/iucABCD*), *sit* (*sitABCD*) and salmochelin (*iroBCDEN*) operons. All three operons encode high-affinity iron-transport systems that are used by bacteria to obtain iron in low-iron conditions such as those they encounter in host fluids and tissues. These operons have previously been reported in virulence plasmids of UPEC, APEC and NMEC with high frequency and have been associated with ExPEC virulence [34]. The data indicate that the three iron uptake systems, namely the ferric aerobactin system, the salmochelin siderophore system, and sitABCD system, have been associated with ExPEC virulence, and are encoded by core the genome of NMEC large virulence plasmids [35]. Some of APEC’s VGs are located at the plasmids typical of APEC strains, for example: IncFIB, IncFIC, pTJ100, ColV lub ColBM [6, 36].

Another gene found in the core genome of NMEC large virulence plasmid is *iss*. This gene encodes a protein linked with increased serum survival in human *E. coli* isolates. Numerous studies have documented its strong alignment with virulent (but not with avirulent) *E. coli* strains. The genes *ompT* and *hlyF* are also found in the core genome of APEC’s large virulence plasmids. *ompT* is predicted to encode the 42-kDa pro-protein, which is processed in the membrane to the 40-kDa mature form. Mature iss functions as a narrowly specific outer membrane endoprotease that has three functions: (1) it cleaves paired basic residues and is involved in membrane protein turnover, (2) can degrade interferon-gamma in vitro, (3) cleaves the human defensin LL-37. The *hlyF* gene is predicted to encode the putative hemolysin gene [34, 37]. Core genes of ExPEC-like plasmids in NMEC are listed and presented in Table 4.

**Table 4:** Core genes of ExPEC-like plasmids in NMEC

Gene names	Description
*Iron uptake systems*	
* iroB*	Putative glucosyltransferase
* iroC*	ATP binding cassette ABC transport homolog
* iroD*	Putative ferric enterochelin esterase
* iroE*	Putative hydrolase
* Iron*	Outer membrane receptor fepA
* iucA*	Aerobactin siderophore biosynthesis protein
* iucB*	N(6)-hydroxylysine acetylase
* iucC*	Aerobactin siderophore biosynthesis protein
* iucD*	l-lysine 6 monooxigenase
* iutA*	Ferric aerobactin receptor precursor
* sitA*	Iron/manganese transport protein, periplasmic-binding protein
* sitB*	Iron/manganese transport protein ATP-binding component
* sitC*	Iron/manganese transport inner membrane component
* sitD*	Iron/manganese transport protein, inner membrane component
* ToxinhlyF*	Hemolysin
*Resistance to innate immunity*	
* ompT*	Outer membrane protease
* Bor*	Bacteriophage lambda bor protein

The purpose of this study was to characterize *E. coli* strains isolated from different sources—humans, animals and food in the context of the presence of bacterial genes encoding virulence factors responsible for the production of adhesins, toxins and iron acquisition systems (siderophores) in relation to the growing number of infections with ExPEC etiology.

#### The role of surface virulence factors in the development of ExPEC infections

The ability of bacteria to adhere to host cells is a necessary condition allowing bacterial pathogens to colonize the body. This phenomenon is referred to as tissue tropism and involves specific interaction with the target receptors and the surface of a particular tissue [38].

### Adhesive properties of ExPEC

Many surface structures play a significant role in the process of specific adhesion. There are three main types of adhesins: fimbriae, afimbrial adhesins (Afa), and outer membrane proteins (OMPs) [39]. The expression of surface adhesins increases the virulence of pathogenic *E. coli* by initiating close contact of the bacteria with the host cell wall. Most genes that determine the presence of fimbriae on the surface of bacterial cells are encoded chromosomally or, less frequently, within the plasmid DNA. Different bacterial adhesins are adapted to colonize a specific niche. S fimbrial adhesins (*sfa*), F1C (“pseudotype I”) fimbriae (*foc*), coding P-like pili, *papC*, and Iha (*iha*) are the most frequently detected adhesins among isolates from UTI patients [40]. In UPEC strains, receptors for P- and S- fimbriae are located on the surface of epithelial cells lining the host urinary tract [41]. S-fimbrial adhesins are virulence factors present in strains responsible for meningitis and sepsis. These fimbriae have the ability to bind extracellular matrix components and sialoglycoproteins on brain capillary endothelial cells. NMEC contains K1 capsular antigens (*kpsM, neuA*) or the *ibeA* invasion gene. The relationship between the type of infection and the presence of characteristic virulence factors has been demonstrated, e.g. IbeA protein has surface receptors on brain capillary endothelial cells and allows pathogens to invade the nervous system [42].

Compared to NMEC, SEPEC strains may show the presence of K2 capsular antigen (*kpsMII*) and P and F17 fimbriae [43]. In contrast to UPEC, the pathogenesis mechanisms of NMEC and SEPEC strains have not been fully elucidated. The comparative analyzes of DNA sequences of ExPEC genomes indicate that dissemination of their virulence factors is very diverse [32, 44]. Although research has been ongoing for many years, specific criteria for classifying *E. coli* strains as ExPEC have not been established yet. According to results obtained by Johnson et al., ExPECs were defined as *E. coli* isolates containing two or more virulence markers, which were determined by multiplex PCR reaction, including *papA* genes (structural subunit of P-fimbriae) and/or *papC* (P fimbriae), *sfa/foc* (S and F1C fimbriae subunits), *afa/dra* (adhesins binding antigen Dr), *kpsMT* II (group 2 capsular polysaccharides) and *iutA* (aerobactin receptor) [45]. Uropathogenic virulence factors are often detected in UPEC, but they are non-existent or rare in *E. coli* strains of the normal intestinal microflora. The prevalence of gene encoding adhesins in commensal strains and in UPEC has been defined according to studies by Qin et al. P-type fimbrial genes have been reported in 28% of UPEC isolates and in 5% of commensal strains. Genes encoding afimbrial adhesins Afa have been present in 36% of UPEC isolates, but they have not been reported in any of the studied commensal strains [46]. Symptomatic UTIs caused by *E. coli* are associated with the ability of these strains to produce a number of virulence factors, with adhesins as essential virulence determinants [47]. Horizontal gene transfer plays a role in the spread of virulence genes located in mobile genetic elements isolated from strains contaminating different food products. In UPEC strains isolated in Mexico from unpasteurized cheeses, *fimA*-*agn43* was the most frequently detected combination of virulence genes (in up to 29%) [48]. Similarly, Aslam et al. [49] reported the high prevalence of many virulence genes (*ompT*, *traT*, *uidA*, *vat*, *hemF*, *iss* and *cvaC*), including the genes responsible for adhesion, *fimH* and *kpsMT KII* in ExPEC isolates from frozen poultry meat.

### Fimbrial adhesins from ExPEC

Fimbriae with affinity for structures containing mannose residues were classified as type 1, and mannose resistant fimbriae classified as type 2 (e.g. P, S, Dr fimbriae). *FimB* and *fimE* are responsible for controlling the expression of type 1 fimbriae. Three other genes, i.e. *fimF, fimG* and *fimH,* are involved in the adhesive property and longitudinal regulation. FimH adhesin, which is formed from FimA protein subunits, binds to uroplakin 1A receptor (UP1a) of bladder epithelial cells, allowing invasion and formation of biofilm-like intracellular structures [50, 51]. In the mouse model, it has been confirmed that type 1 fimbriae enable bacterial growth in the form of biofilms [47]. Moreover, Tarchouna et al. [52] have examined the role of *E. coli* virulence factors in the UTI pathogenesis. The prevalence of genes coding for fimbrial adhesive systems has been 68% for *fimH*, 41% for *pap*, and 34% for *sfa/foc*, whereas afimbrial adhesins (*afa*) have been detected in 20% of the examined strains. Similarly, high prevalence of adhesin-encoding genes in UPEC isolates has been reported by Rahdar et al. [53], namely 95% for *fim*, 57% for *pap*, 16% for *foc*, and 81% for *sfa*.

Mannose-resistant fimbriae include hemolytic F-type fimbriae, which are encoded by 11 genes in the *pap* genes cluster located on the chromosome. PapG adhesin occurs in 3 molecular variants: PapGI, PapGII, PapGIII, with PapG III allele associated with bladder inflammation in women and children, and PapGII related to human bacteremia. The P fimbriae are also common virulence factors in renal transplant patients and in patients with acute renal impairment [47, 54]. According to Shetty et al. [55], high prevalence of adhesin-encoding genes from *E. coli* isolates in patients diagnosed with UTI confirms that these structures are necessary to cause an infection. It is worth noting that in the group of strains with adhesin-coding genes, two genes conditioning fimbrial production, i.e. *pap* and *sfa*, have been found in 30.43% of isolates. In another study, vegetable samples contaminated with sewage from India, Thailand, Vietnam, and the Dominican Republic were analysed (Table 5). The authors have detected one or more uropathogenic virulence genes in 17 out of 26 (65.4%) *E. coli* strains isolated from local vegetables, which suggests that although the presence of a single virulence gene is not sufficient to classify the strain as UPEC, it is worth paying more attention to non-animal food products as the route for the spread of virulence genes. The simultaneous analysis of the various potential UPEC/ExPEC reservoirs may be useful in assessing potential risk factors for infections and help better target research to suppress the spread of pathogenic *E. coli* strains.

F1C fimbriae (*foc*) can bind β-GalNac-1, 4b-Gal residues on glycolipids expressed by epithelial cells of distal tubules and cells of the collecting ducts of the kidney, as well as by endothelial cells of the bladder and kidneys [41]. F1C fimbriae are expressed by about 14% of UPEC isolates and are associated with S-type fimbriae (*sfa*). These two adhesins show a high degree of homology, but they differ in receptor specificity; S fimbriae are encoded by 9 genes. The *sfaA* gene contains information about the major subunit, and the *sfaS* gene - information about specific adhesion, which binds to the α-sialyl-(2,3)-β-Gal receptor in the renal tubular epithelial cells, renal glomeruli, or vascular epithelium. SfaS adhesin produced by the S fimbrial adhesion (*sfa*) mediates interactions with sialic acid receptors on renal epithelial and endothelial cells. S fimbriae allow invasion of pathogens to host tissues, as they are often detected in strains responsible for meningitis and ascending UTIs, including pyelonephritis or sepsis [47, 56]. In turn, the plasmid-encoded MrkD protein is characteristic for type 3 fimbriae adhesin, which binds to type V collagen in renal tubules and contributes to the formation of bacterial biofilm and colonization of urological catheters [57]. Szemiako et al. [58] have analyzed genetic determinants of virulence of *E. coli* strains, which enable bacterial translocation from the urinary tract into the bloodstream in renal transplant patients. The authors have analyzed the following genetic determinants of adhesins: *fimG/fimH* (type 1 fimbrial genes), *sfaD/sfaE* (S fimbrial genes), *papC* (P fimbrial gene), and genes encoding fimbriae of the Dr family, afa/dra (B-C). The results obtained by the researchers indicate that the combination of genes encoding two adhesion factors simultaneously, e.g. P + Dr, P + S, or S + Dr, S + fim, is associated with a much greater risk of such a strain entering the circulatory system. In another study, Krawczyk et al. [56] have analyzed *E. coli* strains which are capable of translocating from the gastrointestinal tract into the vascular bed. The examined bacterial strains were isolated from clinical material collected from 115 patients with hematopoietic tumors and bacteremia. Thanks to the methods of genotyping used in the research, the authors have reported that 89 *E. coli* strains isolated from blood have the same genotype as *E. coli* isolated from intestines. A detailed genetic analysis of the examined strains has indicated that ampicillin-resistant *E. coli* strains with the *afa/dr* pattern are most commonly associated with bacteremia. The authors have indicated that the coexistence of *pap*C, *sfa*, *usp* and *cnf1* genes encoding virulence factors may predispose *E. coli* to translocate from the gastrointestinal tract to the vascular bed in the group of patients with hematological cancers.

### Afimbrial adhesins from ExPEC

An important group of superficial virulence factors associated, among others, with the occurrence of pyelonephritis and recurrent cystitis is the Afa/Dr family of adhesins, which contains both the fimbrial adhesins, with Dr fimbriae and Afa afimbrial adhesins found mainly in uropathogenic *E. coli* strains [59]. *draE/afaE* determine the expression of genetic information and the production of adhesins, and *draA*, *draB*, *draC*, *draD* (*afaA, afaB, afaC, afaD*) genes encode helper proteins. The receptor common for the whole family is the decay-accelerating factor (DAF), expressed on the surface of erythrocytes and cells of other tissues (e.g. the epithelium of the urinary tract). Adhesin Dr has two other receptors. One of them is basement membrane type IV collagen, an important factor favoring UTIs, and the other one is carcinoembryonic antigen related to cell adhesion molecules (CEACAM), which serves as a signal receptor regulating physiological changes. Attachment to this receptor facilitates bacterial invasion of intraepithelial cells [60].

### The role of capsules and biofilm in the adhesion of ExPEC strains

The coating on the outer surface of the cell wall protects bacteria against phagocytosis and the bactericidal action of the complement system. The diversified structure of polysaccharide capsules produced by several dozen types of UPEC strains allows mimicking host tissue components and making recognition by the immune system difficult [47]. *E. coli* strains expressing the K1 antigen are associated with the development of neonatal sepsis [61]. The production of the capsular K1 antigen is contingent on the presence of the *neuC* gene. The group of 14 *kps* operons is responsible for the K1 capsule formation. The *kpsMTII* gene encodes proteins required for polymer translocation from its site of synthesis to the cell surface. Wijetunge et al. [26], based on the genotyping results of the examined NMEC strains, have found that all isolates have the capacity to invade human brain endothelial cells, and more than 70% of them carried *kpsII,* K1, *neuC, iucC*, *sit* genes. The examined NMEC strains demonstrated very high (79.2%) ability to form biofilms.

Bacterial capsular antigen K that covers the cell surface may inhibit adhesion to epithelial cells. The interaction of FimF with d-mannose inhibits the transcription of capsid genes, which may lead to a reduced amount of K antigen on the surface of the *E. coli* cell, and, as a result, facilitate the adhesion process [62]. In turn, UpaG, a member of auto-transporter family of adhesins, shows affinity for fibronectin and laminin, allowing UPEC adherence to the bladder epithelium. Additionally, UpaG participates in creating biofilm on plastics, which facilitates colonization of urological catheters [63].

The translocation of bacterial cells from the growth phase in plankton to the growth phase in the biofilm is associated with a change in the expression of many genes encoding not only virulence factors, but also regulatory proteins. The biofilm-forming ability of *E. coli* is affected by the expression of gene encoding antigenic Agn43 (i.e. *flu*), which is involved in aggregation of *E. coli* cells [64]. Urinary tract infections can often cause bacteremia, especially in hospitalized patients, when, due to catheter contamination, biofilm is produced by invasive ExPEC strains possessing many virulence factors [65–67]. In their analysis of genes determining production of adhesins by SEPEC strains, Conceição et al. [18] have indicated that 98.0% of these SEPEC strains tested positive for the *fimH*, 69.4% for *flu*, 53.1% for *csgA*, 38.8% for *vat*, and 32.7% for *iha*. The authors have suggested that SEPEC adhesion to cell surfaces occurs with the involvement of non-*fimH* mechanisms.

### Prevalence and transmission of ExPEC strains in different food products

There are many studies on the reservoir of ExPEC in relation to food production and distribution [7, 49, 68, 69]. Examples of *E. coli* (ExPEC phenotype) strains with high prevalence of virulence genes detected from different food products are presented in Table 5. In 2015, using multiplex PCR test, Mitchell et al. [70] examined *E. coli* strains isolated from chicken meat and egg shells determining 5 ExPEC-defining markers, i.e. *papA* and/or *papC* (P fimbriae), *sfa* and/or *foc* (S and F1C fimbriae), *afa* and/or *dra* (Dr-binding adhesins), *kpsM II* (group 2 capsule), and *iutA* (aerobactin system). In the initial stage of the study, the authors have established genotypic and phenotypic criteria characteristic for individual pathotypes in the ExPEC group, which allowed assigning examined isolates to a specific pathotype. These included the culture in the urine for UPEC, while NMEC was determined by the detection of two genes, *kpsMTK1* and *ibeA*. Based on the obtained results, it has been found that the prevalence of ExPEC is much lower among egg-source isolates than in the group of chicken-source *E. coli* isolates (4.7% and 21% respectively). Many methods have been applied to identify the ExPEC strains with the zoonotic potential, including strains able to cause one or more diseases in the animal models of sepsis, meningitis or UTI [5, 22, 71, 72]. This confirms the results of other studies which have proven that *E. coli* isolates from the feces of healthy Danish broiler chickens were virulent in the UTI mouse model [73]. APEC share common virulence factors with UPEC, which confirms earlier hypotheses stating that ExPEC play a role in food-borne infections and pointing to the association of APEC with extraintestinal infections in humans [28, 74, 75]. APEC strains could be carried from birds to human by improperly prepared poultry meat and by direct contact with birds and their feces [76, 77]. Borzi and colleagues showed that helmeted guinea fowl *(Numida meleagris)* could be a reservoir of antibiotic resistant APEC what is a potential health risk for other species including humans [78].

**Table 5:** Examples of *E. coli* (ExPEC phenotype) strains detection from different food products

Localization/Country	Origin of food products	*E. coli* (ExPEC phenotype) with high prevalence of virulence genes	References
Canada	Frozen poultry meat	*fimH*, *kpsMT KII*	Aslam et al. [49]
Imported to Switzerland from India, Thailand, Vietnam, and the Dominican Republic	Vegetable samples, retail poultry meat	*traT, fyuA, chuA, vat*	Müller et al. [118]
United States	Chicken meat and egg shells	*ompT*, *iss*, *traT*, *fimH, hra, papA, papC i papEF*	Mitchell et al. [70]
Mexico	Unpasteurized cheeses	*fimA-agn43,fyuA*	Guzman-Hernandez et al. [48]
Egypt	Dairy products: raw milk, Karish cheese, Ras cheese	*hlyA, cnf, vat, fyuA, iroN, iutA*	Ombarak et al. [69]
China	Pork samples	*kpsMII*, *fimH*, *papC*, *sfaS*, *focG*, *fimH*, *afa,iutA,ireA,cnf,*	Khan et al. [80]
Brasil	Poultry	*pap, sfa, usp*	Cunha et al. [81]

The acronym FUTI (food-borne urinary tract infection) describes UTIs associated with contaminated food. Pathotypes of *E. coli* responsible for FUTI have not yet been precisely defined [74]. Jakobsen et al. [79] have attempted to detect the presence of ExPEC-associated virulence genes in *E. coli* isolated from UTI patients, production animals, and meat. All isolates have been tested for the presence of eight ExPEC-related genes (*kpsMII, papA, papC, iutA, sfaS, focG, afa, hlyD*). The obtained results suggest that food-producing animals may be reservoirs of strains carrying ExPEC-related virulence genes responsible for UTI in humans. A detailed analysis of the genetic virulence determinants and resistance profiles to antimicrobial agents in the examined ExPEC strains isolated from UTI patients, production animals, or meat, have shown their high similarity, which in turn may increase the risk of zoonotic infections caused by these strains. Khan et al. [80] have observed that in the group of ExPEC strains isolated from pork samples in Hubei (China), up to 85% belonged to group B2. Among genes encoding surface virulence factors, the most prevalent genes were *kpsMII* (74.5%), *fimH* (70.4%) and *papC* (47.3%), while among much less frequently detected strains in group D, the most widespread virulence genes were *sfaS* and *focG* (76.9%), *fimH* (46.2%), and *afa* (38.5%). The authors suggest that, similarly to the avian or human ExPEC, *E. coli* strains isolated from pigs have many genetic determinants of virulence, which promote UTI in humans.

In the study conducted in Brazil, Cunha et al. [81] have characterized 27 APEC isolates from different poultry farms that belonged to serogroup O6, which has often been isolated as the etiological agent of UTI and sepsis, not only in Brazil, but also globally. These strains have many genetic virulence determinants that commonly occur in UPEC and SEPEC isolates, and, to a lesser extent, in NMEC isolates obtained from newborns with meningitis, in which adhesin-encoding genes were most frequently detected: *pap* (85%), *sfa* (100%). On the other hand, Canadian studies on the frequency of detecting ExPEC in meat, have reported a significantly higher share of these isolates in samples from chickens compared to beef or pork [82]. The genetic similarity between *E. coli* strains isolated mainly from chickens and ExPEC that cause UTI in humans may indicate that meat, particularly from chickens, may be a reservoir for ExPEC causing UTI in humans. Many researchers suggest that detection of *E. coli* with virulence factors that facilitate the development of human ExPEC infections in food products of animal origin, particularly in poultry, is currently an important problem of food safety that requires constant monitoring [83].

Due to the phenomenon of phase variation, i.e. controlling the expression of bacterial structures responsible for adhesion, bacterial cells can regulate the process of developing fimbriae. Phase variation protects pathogens from the immune system. No fimbriae expression can result in the absence of adherence mechanisms and be a signal to produce other types of adhesins. It has been observed that strains without typical adhesins could adhere to and invade T-24 cells, and induce infections such as strains with those adhesins [83]. The results of the experiment show that the adhesion process is very complex and many mechanisms remain unexplained.

#### ExPEC siderophores and toxins

VFs related to the pathogenicity of ExPEC are numerous and have a wide range of activities, from those related to bacteria colonization to those related to virulence, i.e. invasiveness. Current scientific evidence suggests that infection cannot be caused by a single virulence factor, but by many specific agents. Most importantly, preference of bacteria to colonize a specific site in the host organism is associated with timed expression of genes encoding virulence factors. Additionally, it has been observed that the coexistence of many virulence genes is associated with increased intestinal translocation [3, 49, 70], and VF profiles of ExPEC strains from bacteremia are highly diversified [3]. Therefore, an interesting research trend is to determine the relationship between individual VFs typical of ExPEC and the localization of extraintestinal infection [70]. Siderophores, described extensively in this section, are secondary metabolites, with their primary function being to assist in capturing iron to maintain bacterial growth and development [84]. Although siderophore production can aid bacterial growth, production of these factors may increase metabolic costs [85]. Consecutive VFs, which were also the focus of this part of the paper, are toxins. Toxins play a very important role during an infection as they contribute to the spreading of bacteria in tissues, increase cytotoxicity and insensitivity to neutrophils [86].

### Iron in human body

Iron (Fe) is essential for life and proper functioning of all living organisms, including vertebrates, invertebrates, and prokaryotic organisms such as bacteria [87]. Iron, as an electron carrier, plays a key role in basic cellular processes such as cellular respiration, DNA replication, or oxygen transport. Moreover, iron is built into the protein structure as a prosthetic group [88, 89]. To properly promote metabolic processes, share and display pathogenic properties, bacteria need access to Fe [90], and gaining such access is difficult as only a small amount of iron in nature is available to organisms [87, 91]. In the host organisms, iron is sequestered or distributed within cells and tissues by binding to proteins storing iron in tissues (ferritin). In higher organisms, iron mainly occurs as trivalent ions (Fe^+3^) of very low solubility [92]. Therefore, Fe in the porphyrin ring is a part of heme—cofactor of hemoglobin, myoglobin, and cytochromes [90, 93]. Iron ions stored in such a way become inaccessible to bacteria. Another strategy of preventing bacteria from using Fe resources in higher organisms is that it is captured by serous transferrin, which has very high affinity for Fe. When bacteria enter the body, macrophages accumulate free Fe in the cytoplasm [87, 94]. It is estimated that the concentration of unbound Fe in human serum is 10^−24^ M, whereas in tissues this value varies from 10^−7^ M to 10^−5^ M (M = mol/dm^−3^). These values are significantly lower than the level of Fe required for the proper functioning and division of bacterial cells, which ranges from 10^5^ M to 10^6^ M [95–97].

### Acquiring iron by ExPEC

Iron deficiency weakens bacterial adaptive abilities by generating disturbances in cellular capsules [98]. However, thanks to their adaptive abilities, bacteria have developed mechanisms that enable them to acquire iron in the host body, thus survive and cause infection [99]. In the case of ExPEC strain, access to Fe in the blood serum is of paramount importance. Since *E. coli* can cause sepsis and infections of various organs with very low iron availability, this pathotype has developed many strategies for obtaining iron from infected sites [100, 101]. It has been proven that ExPEC strains have membrane pumps that transfer Fe to the cell interior, e.g. the FeoAB pump found in commensal and pathogenic *E. coli* [102], SitABCD transporter (ABC type transporter, ATP-binding cassette) [103], and Hma and ChuA transporters that enable Fe uptake directly from extracellular heme [96]. ExpEC pathotypes have specialized systems to indirectly uptake Fe based on the so-called shuttle mechanism involving small molecules (200–2000 Da) with high affinity for Fe ions (strong chelating properties) [97]. Interestingly, these complexes, called siderophores, occur not only in prokaryotes, but also in fungi and plants. Siderophores allow capturing trivalent iron (Fe^+3^) from ferritin and transferrin [89, 96]. The important role of siderophores in ExPEC virulence may be confirmed by the results obtained by Stromberg et al. [73] who have reported 100% gene prevalence for these factors in 40 ExPEC isolates from chicken faeces, including 38% strains growing in human urine.

### Types of siderophores in ExPEC

Siderophores can be divided into five main classes: catecholates, phenolates, hydroxamic acids, α-hydroxycarboxylates, and a mixed type containing different siderophores. Extraintestinal pathogenic *E. coli* are equipped with siderophores that increase their virulence: enterobactin and salmochelin (catecholate siderophores), yersiniabactin (phenolate siderophore), and aerobactin (a mixed-type siderophore). Since the siderophore enterobactin is found in virtually all *E. coli* strains, both commensal and pathogenic, a question arises whether it should be considered as a virulence factor. Enterobactin and salmochelin are synthesized not only by *E. coli*, but also by other pathogenic gut bacteria such as *Salmonella* spp. or *Klebsiella* spp. [96, 97]. Salmochelin, a glycosylated derivative of enterobactin, was first discovered in *Salmonella* spp. (hence the name of this siderophore). Salmochelin occurs in ExPEC strains and is considered a characteristic virulence factor for the ExPEC pathotype. Salmochelin is encoded by the *iroBCDEN* gene cluster [104] located on ColV or ColBM virulence plasmids, or identified on PAIs [105]. It has been reported that *IroB* is the sole gene with glycosyltransferase activity necessary for salmochelin production, which leads to glycosylation of enterobactin that changes its properties from strongly hydrophobic to hydrophilic. This change may contribute to the virulence of ExPEC [106, 107]. The *iroN* gene is an ExPEC salmochelin marker and an important virulence gene in virotyping ExPEC strains [108]. Aerobactin is another siderophore characteristic for ExPEC. Similarly to salmochelin, this siderophore is also encoded by ColV and ColBM virulence plasmids of ExPEC [96, 105]. Aerobactin receptor shows much greater efficiency in capturing Fe than enterobactin. Extraintestinal pathogenic *E. coli* may include another type of siderophore, i.e. yersiniabactin, which was originally detected in *Yersinia pestis*, mainly during colonization of the urinary tract. Yersiniabactin contributes to the pathogenicity of uropathogenic *E. coli* (UPEC), especially during colonization of the urinary tract. Yersiniabactin may protect bacterial cells against host immune response [88, 96, 109].

### Toxins produced by ExPEC

The analysis of literature data allowed extracting the most frequently detected genes encoding toxins in ExPEC, which include: *tsh* (temperature-sensitive hemagglutinin tsh autotransporter), *hlyA, hlyD i hlyF* (α-hemolysin)*, cnf1* (cytotoxic necrotizing factor 1), *sat* (secreted autotransported toxin), *pic* (protease involved in colonization), *vat* (vacuolating autotransporter protein), *cdtB* (cytolethal distending factor), and *astA* (enteroaggregative *E. coli* toxin) [2, 44, 69, 70, 110–112]. Genetic criteria proposed by Mellata et al. in studies on sepsis, meningitis, and rodent models of UTI have defined NMEC based on the presence of, among others, one or more genes encoding toxins: *hlyF, tsh, astA,* and *cdtB.* Depending on strains identified as NMEC, the authors have detected the *tsh* or *cdtB* gene, two genes (*hlyF, tsh)* or three genes (*astA, hlyF, tsh*). A similar correlation was observed in the case of siderophore genes, i.e. *fyuA, iroN, iutA, ire*, which were present in UPEC and NMEC (two or more) [70, 83]. In genotyping tests, studying ExPEC pathogenicity for poultry and rodents, Stromberg et al. have used multiplex PCR for the above-mentioned genes [73]. A comparative analysis using multiplex PCR has shown that ExPEC isolates from birds and humans contain similar sets of VF-encoding genes, and they fall into the same phylogenetic groups (A, B1, B2, and D), which suggests the zoonotic origin of ExPEC [113]. Chinese studies have indicated a dominating B2 group (169/285) among 285 analyzed samples from pig farms, slaughterhouses, and supermarkets. Most frequently detected genes for *E. coli* toxins and siderophores were *iutA* (82.9%, 97/117), *ireA* (60.6%, 71/117), *cnf* (57.3%, 67/117), *hlyD* (30.7%, 36/117), *fyu* (21.4%, 25/117), and *vat* (9.4%, 11/117), which represented more than half of all the virulence genes detected in *E. coli* strains [80].

### Prevalence of genes encoding toxins and siderophores in ExPEC food isolates

In recent years, extensive research has been conducted into the spread of ExPEC in the human population in the context of eating contaminated food [47, 67, 82, 110, 113, 114]. As it has already been pointed out, detecting of these pathogens in food products is difficult because specific molecular markers have not yet been identified for ExPEC [73]. Mitchell et al. [70] have characterized and thoroughly analyzed ExPEC isolates from food products taking into account their division into UPEC, NMEC and SEPEC pathotypes. The authors have suggested that poultry can be the source of virulent pathotypes, capable of inducing UTI, neonatal meningitis, and sepsis. In the Canadian research, ExPEC isolates have been reported as the microbiological contamination of poultry available for sale in retail outlets, with a frequency of 8.4%. In all genetically analyzed strains, out of 54 virulence genes, 9 dominated in the case of ExPEC, while *vat*, responsible for the production of vacuole toxin, was the most frequently detected gene encoding toxins [28]. Studies carried out in Mexico have shown that the popular, region-specific, unpasteurized cheeses such as “Queso Fresco”, “Queso Crema”, “Queso Doble Crema”, “Queso Panela”, and “Queso Poro” were microbiologically contaminated with *E. coli* in more than 50% of cases. Although the highest percentage of strains belonged to the group causing gastrointestinal symptoms, 26% of strains were potential UPEC. Genes determining the synthesis of toxins and siderophores (*hlyA*, *vat, cnf1, fyuA, iroN, iutA)* have been used to identify ExPEC strains. The *fyu*A gene was found in 39% of isolates, and *iutA* and *hlyA* genes were detected in less than 10%. Potential uropathogenic *E. coli* (UPEC) were isolated in 29% of samples. The general hygiene conditions and practices of producers influenced the quality of cheese, which was reportedly quite low [48]. Egyptian research from 2016 indicated that raw milk, Karish cheese and Ras cheese were highly contaminated with *E. coli* carrying *hlyA, cnf1, vat* genes and *fyuA, iroN, iutA* siderophores [69]. Moreover, eggs are reportedly contaminated with *E. coli* that cause diarrhea and with ExPEC pathogens. The Spanish study, based on data from 2016, shows that eggs for retail sale were of good quality due to the low incidence of *E. coli* on egg surface (34/180), which was around 19% [97]. In the United States, ExPEC pathotypes were detected only in 4% of examined eggs, and in 96% of poultry [70].

The time shift between colonization of ExPEC and the development of infection remains problematic in the context of establishing the relation between consumption of contaminated food and the appearance of the first symptoms of the disease. The most difficult part is to gather evidence in order to prove that ExPEC cause disease symptoms and to examine the mechanism of transition from the asymptomatic colonization of intestines to the spread outside the digestive tract. A significant challenge faced by researchers attempting to attribute ExPEC transmission to food, people or the environment is the delay between ExPEC colonization and infection [115]. Contrary to *E. coli* that cause diarrhea with symptoms occurring shortly after ingestion, ExPEC can live in human intestines for months or even years until they find favorable conditions, primarily associated with impaired immune function, urinary catheters, urethral infection, or prostate biopsy [116].

### Pathogenicity of ExPEC resulting from toxins and siderophores

In a retrospective study of *E. coli* O45 isolates recovered from patients with colibacillosis and from poultry, Spanish and French researchers have detected multiple clusters of PFGE patterns similar at < 85% [67]. Direct transmission of ExPEC from food to humans is difficult to detect. Moreover, it would be highly unethical to test the effectiveness of causing ExPEC infections in humans. Therefore, the only possible solution is to determine the pathogenicity of bacteria on animal models or human cell lines. In the Stromberg et al.’s research from 2017 [73], it has been assessed whether chicken fecal *E. coli* isolates from healthy chickens could cause colibacillosis in chicken and rodent models. Selected isolates, genetically identified as ExPEC (showing i.a. *hlyF*, *iutA*, *tsh* genes) were tested for their virulence potential for humans to induce sepsis, meningitis, and UTIs in female BALB/c mice and CBA/J mice (to cause only UTI). It has been shown that the examined *E. coli* strains have the ability to cause central nervous system infections in the Sprague–Dawley rats meningitis model. Interestingly, some isolates classified as non-ExPEC were able to cause ExPEC-associated illnesses in animal models but the authors do not explain that mechanism. Focusing on the virulence factors determining the development of UPEC infections, Hagan et al. [40] have conducted a very interesting analysis of gene expression in *E. coli* isolates from the urine of women with suspected UTI. An important element was to link the expression of individual genes with the process of infection, which was assumed to represent naturally occurring processes in the cells during infection in humans. The authors have suggested that during UTI, *E. coli* uses many iron and carbon uptake pathways, which allows intensive replication with simultaneous inhibition of type 1 fimbriae synthesis. In another study, 96 ExPEC strains isolated from the urine and blood of patients at the University Hospital of Londrina have been compared with 50 commensal strains from healthy individuals. Among 96 patients infected with ExPEC, 90 (93.8%) were suffered from UTIs that were classified as cystitis (91.1%), urosepsis (6.7%), and pyelonephritis (2.2%). The *iroN*, *iutA*, *ompT* and *hlyF* genes have been more frequently detected in clinical ExPEC isolates compared to commensal isolates [117]. Other studies have demonstrated that strains isolated from cases of avian colibacillosis are able to cause disease in a rat model of human neonatal meningitis [5]. In order to further evaluate the zoonotic potential of ExPEC, the virulence of selected strains has been assessed in the mouse model. The authors have analyzed the potential of ExPEC to cause sepsis, meningitis and UTI, and whether virulence factors detected in vitro translate to virulence factors in vivo. Certain *E. coli* strains isolated from chickens were able to cause one or even many diseases, but interestingly, not only from the ExPEC spectrum. It has been assumed that *E. coli* may use different virulence-related strategies to induce sepsis, besides these associated with resistance to bactericidal action of complement [83]. These two examples prove that there is a need for further research on pathogenic *E. coli* in order to explain their virulence mechanisms.

## Summary

Taking into account the latest epidemiological data on ExPEC infections, large-scale molecular research should be carried out on new reservoirs and pathways of ExPEC strains, and, in particular, on the mechanisms underlying invasive diseases and asymptomatic intestinal colonization so as to guide the development of different prophylactic procedures, such as production of vaccines or therapeutic strategies targeting new bacterial agents. This also underlines the importance of educating producers, traders and sellers about the new emerging microbial threats and the significant role of proper food trade to minimize such risks. A comparative analysis has shown that avian and human *E. coli* isolates contain similar sets of genes encoding virulence factors, and that they belong to the same phylogenetic groups, which may indicate the zoonotic origin of ExPEC. Many authors confirm the presence of genetically closely related strains isolated from infections of epidemic character, which usually presented unusual virulence profiles or antibiotics susceptibility. Researchers are particularly interested in the problem of food contamination by ExPEC/UPEC strains in correlation with their virulence factors. With the increasing demand for poultry meat and poultry products and the growing poultry industry around the world, food safety is an important challenge for public health. In order to assess the dissemination of ExPEC strains we should examine the level of genetic similarity between isolates from different hosts. Multiple levels of genotyping are proposed, in which typing of strains, plasmids and genes is compared in order to obtain a more complete picture of this complex problem.

## Abbreviations

UTI: urinary tract infection
HAP: hospital-acquired pneumonia
SSI: surgical site infection
HUS: hemolytic-uremic syndrome
ExPEC: extraintestinal pathogenic *E. coli*
VF: virulence factors
UPEC: uropathogenic *E. coli*
NMEC: neonatal meningitis *E. coli*
SEPEC: sepsis-associated *E. coli*
APEC: avian pathogenic *E. coli*
PAI: pathogenicity island
ABU: asymptomatic bacteriuria
OMPs: outer membrane proteins
DAF: decay-accelerating factor
CEACAM: carcinoembryonic antigen related to cell adhesion molecules
FUTI: food-borne urinary tract infection
